# Expression of Adenoviral E1A in Transformed Cells as an Additional Factor of HDACi-Dependent FoxO Regulation

**DOI:** 10.3390/cells9010097

**Published:** 2019-12-30

**Authors:** Alisa Morshneva, Olga Gnedina, Tamara Marusova, Maria Igotti

**Affiliations:** Institute of Cytology, Russian Academy of Sciences, 194064 St. Petersburg, Russia; 1195alisa@gmail.com (A.M.); olga.o.gnedina@gmail.com (O.G.); tamaramarusova@gmail.com (T.M.)

**Keywords:** FoxO, E1A, cancer, apoptosis, histone deacetylase inhibitor (HDACi)

## Abstract

The adenoviral early region 1A (E1A) protein has proapoptotic and angiogenic activity, along with its chemosensitizing effect, making it the focus of increased interest in the context of cancer therapy. It was previously shown that E1A-induced chemosensitization to different drugs, including histone deacetylases inhibitors (HDACi), appears to be mediated by Forkhead box O (FoxO) transcription factors. In this study, we explore the relationship between E1A expression and the modulation of FoxO activity with HDACi sodium butyrate (NaBut). We show here that the basal FoxO level is elevated in E1A-expressing cells. Prolonged NaBut treatment leads to the inhibition of the FoxO expression and activity in E1A-expressing cells. However, in E1A-negative cells, NaBut promotes the transactivation ability of FoxO over time. A more detailed investigation revealed that the NaBut-induced decrease of FoxO activity in E1A-expressing cells is due to the NaBut-dependent decrease in E1A expression. Therefore, NaBut-induced inhibition of FoxO in E1A-positive cells can be overcome under unregulated overexpression of E1A. Remarkably, the CBP/p300-binding domain of E1Aad5 is responsible for stabilization of the FoxO protein. Collectively, these data show that the expression of E1A increases the FoxO stability but makes the FoxO level more sensitive to HDACi treatment.

## 1. Introduction

The early region 1A (E1A) of adenovirus encodes two major proteins E1A 12S and E1A 13S, translated from two alternatively spliced transcripts, including the 12S and 13S messenger RNAs (mRNAs) encoding 243 (243R) and 289 (289R) amino-acid oncoproteins [1]. These proteins are synthesized immediately after viral infection and are predominant in transformed cells. Stable E1A expression leads to immortalization and, in cooperation with the other oncogenes, to complete oncogenic transformation [2]. E1A expression is essential for viral replication, since E1A stimulates progression from the gap 1 (G1) to synthesis (S) phase, allowing the virus to use host cell replication machinery. E1A gene products participate in the regulation of DNA synthesis, transcription, cell immortalization, and transformation [3].

E1A is a promising candidate for cancer therapy, due to its proapoptotic [4], antiangiogenic [5,6], and chemosensitizing activities [7]. E1A is able to enhance the activity of a variety of proapoptotic stimuli, including chemotherapeutic agents and irradiation [8,9,10,11,12,13,14]. It was shown that the sensitizing effect of E1A on histone deacetylases inhibitors (HDACi) (e.g., SAHA, TSA) was stronger than the effect of the other chemotherapeutic drugs tested (5-fluorouracil, cisplatin, etoposide, paclitaxel) [12]. Moreover, the sensitizing effect of E1A on HDAC inhibitors was not observed in the normal cells [10,12]. The absence of increased cytotoxic influence of the E1A/HDACi combined therapy on normal cells makes this combination a promising anticancer approach.

By changing the expression of different sets of genes, HDACis suppress cell growth and induce cell differentiation, senescence, or apoptosis more effectively in transformed cells than in normal cells [15,16,17,18]. In addition to cell growth arrest, HDACis augment the effects of cytotoxic agents traditionally used for tumor therapy [19,20,21]. Due to their marked antiproliferative effect, inhibitors of HDAC are currently being tested in cancer therapeutic clinical trials [16,18,22].

The molecular mechanisms determining the E1A-mediated cellular sensitivity to apoptosis are not yet fully known. However, E1A-dependent chemosensitization appears to be connected with Forkhead box O (FoxO) transcription factors [23]. A knockdown of FoxO3a abolished E1A-induced sensitivity to cytotoxic drugs [23]. Moreover, some of the proteins mediating an E1A-induced chemosensitization of tumor cells to apoptosis are either regulators or effectors of FoxOs transcription factors [12,24,25]. One of the molecular mechanisms of E1A-induced chemosensitization is upregulation of protein phosphatases PP2A [24] or MKP1 [25] that are involved in the dephosphorylation and stabilization of FoxOs transcription factors [26,27,28]. Alternatively, E1A can induce sensitization to anticancer drugs by downregulating the activity of a critical survival factor Akt [29], which was shown to inhibit FoxOs [30,31]. E1A was shown to inhibit ubiquitin-dependent proteolysis of FoxO3a, leading to its stabilization [23], which can be partially attributed to E1A-dependent suppression of Akt [32]. E1A was shown to enhance the proapoptotic activity of HDAC inhibitors through additional activation of FoxO target gene *BIM* [12]. Apart from their other effects, HDACis were shown to affect the activity of Forkhead family proteins O (FoxO), partly through CBP/p300-mediated acetylation of FoxO [33]. This activity of CBP/p300 complex is under control of E1A, since E1A binds the complex, reducing its acetylase activity [34].

FoxOs belong to the Forkhead family of transcription factors sharing the common DNA-binding domain FKH [35]. These transcription factors are to some extent involved in the regulation of key cellular functions, such as oxidative stress response, differentiation, cell death, etc., working as integrators among various signal pathways [36]. There are four human FoxO proteins, having overlapping but still distinct expression patterns: FoxO1, FoxO3, FoxO4, and FoxO6 [37]. The loss of the FoxO transcription factors functions in cancer cells may impair or decrease their abilities to arrest cell-cycle progression and promote apoptosis under genotoxic stress, thereby leading to tumor development [38,39].

In this study, we are dealing with FoxO1—the most studied member of the Forkhead family. The functions of FoxO1 and its role in cancerogenesis and tumor progression are quite sophisticated and context-specific. FoxO1 deletions are lethal due to incomplete vascular development in the embryo [40]. Participation in the process of angiogenesis makes FoxO1 a crucial element of tumor growth and development [41,42]. Under some conditions, FoxO1 can induce drug resistance [43]. At the same time, FoxO1 was reported to inhibit the metastasis process in prostate cancer cells [44] and suppress tumor growth [45].

This study is focused on the stabilizing effects of E1A in relation to FoxO, showing the rise in FoxO level in the presence of E1A and establishing the link between HDACi-induced E1A and FoxO degradation. Taken together, the paper deals with FoxO regulation under the HDACi treatment in relation to E1A expression, and it examines the stabilizing functions of E1A.

## 2. Materials and Methods

### 2.1. Cell Lines

We used rodent embryonic fibroblasts transformed either with pE1A vector coding the early region of human adenovirus type 5 (E1Aad5) in complementation with pSV-ras-gpt coding the cHa-ras carrying mutations at positions 12 and 61 (E1A + Ras) or with *Hind*III-G region of Ad5 viral DNA coding E1A and E1B 19kDa (E1A + E1B). E1A + Ras and E1A + E1B transformed and NIH3T3 normal fibroblasts were cultivated in high-glucose Dulbecco’s modified Eagle’s medium (DMEM) with 10% fetal bovine serum (FBS) and antibiotics. Cells were treated with 4 mM sodium butyrate (NaBut) for 24–72 h (Sigma-Aldrich, St. Louis, MO, USA). NIH3T3 mouse fibroblasts, HEK293 human kidney (embryonic) cells, and HCT116 human colorectal carcinoma cells were obtained from the Russian Collection of Cell Cultures (Institute of Cytology, Russian Academy of Sciences, St. Petersburg, Russia). The adenovirus E1A constructs (12S (wild type (wt)), 12S (RG2) and 12S (YH47)) were obtained from Dr. Elizabeth Moran (Cold Spring Harbor, NY, USA). The hemagglutinin (HA)-tagged full-length p300 was a gift from Elizabeth Wilson (Addgene plasmid # 89094; http://n2t.net/addgene:89094; RRID: Addgene_89094). FHRE-Luc was a gift from Michael Greenberg (Addgene plasmid # 1789; http://n2t.net/addgene:1789; RRID: Addgene_1789).

### 2.2. RT-PCR

The total cellular RNA was isolated with Trizol (Invitrogen, Carlsbad, CA, USA). The RT step was performed with 2 µg of RNA and 1 µg of random hexaprimers. The PCR step was performed in the presence of 100 ng of primers to the complementary DNA (cDNA) of mouse or human genes (*p21/waf1*: 5′–GCCGTGATTGCGATGCGCTCA–3′ and 5′–ACAGCGACAAGGCCACGTGGT–3′; *mnsod*: 5′–GCACTGAAGTTCAATGGTGG–3′ and 5′–ACTGAAGGTAGTAAGCGTGCTC–3′; *bim*: 5′–GCCAAGCAACCTTCTGATG–3′ and 5′–CACTGAGATAGTGGTTGAAGGC–3′; *gapdh* (m): 5′–TGTGATGGGTGTGAACCACG–3′ and 5′–CCAGTGAGCTTCCCGTTCAG–3′; *gapdh* (h): 5′–TCATCAGCAATGCCTCCTGCACC–3′ and 5′–ACAGTTTCCCGGAGGGGCCA–3′). PCRs proceeded for 22–32 cycles: A denaturation step at 950 C for 30 s, an annealing step (55 °C for *mnsod*; 56 °C for *bim*; 58 °C for *gapdh*; 63 °C for *p21/waf1*) for 30 s, and an elongation step at 72 °C for 1 min. PCR products were resolved by electrophoresis in 2% agarose gel. The *alpha-tubulin* gene was selected as an internal control in the PCR assay.

### 2.3. Immunoblotting

For immunoblotting, cells were lysed in a buffer containing 1% NP-40, 0.5% sodium deoxycholate, 0.1% SDS, 20 mM glycerophosphate, 1 mM sodium orthovanadate, 5 mM EGTA, 10 mM sodium fluoride, 1 mM phenylmethylsulfonyl fluoride, and a protease inhibitor cocktail. Proteins were separated by electrophoresis in 10–12% polyacrylamide gel in the presence of 0.1% SDS, transferred onto a membrane (Immobilon P), and probed with appropriate antibodies. As primary antibodies, we used antibodies to Foxo1 #2880 (Cell Signaling, Danvers, MA, USA), E1A (M73) sc-25 (Santa Cruz), pan-Ras #OP40 (Calbiochem, San Diego, CA, USA), and alpha-tubulin T5168 (Sigma). Anti-mouse and anti-rabbit antibodies conjugated with horseradish peroxidase (Sigma) were used as the secondary antibodies. Visualization of membrane-bound proteins was performed by enhanced chemiluminescence (ECL, Amersham Biosciences, Buckinghamshire, UK).

Every protein of interest was analyzed at least three times on different sets of samples. The band density was evaluated using ImageJ (1.51q-1, Bethesda, MD, USA). Then, density values were scaled to load control and converted to relative units. The figure plots represent the mean values of several experiments; error bars indicate the standard error of the mean (SEM).

### 2.4. Transfection and Analysis of Luciferase Activity

For transient and stable transfection, cells were plated in 96-well plates (antibiotic-free DMEM with 10% FBS) at a seeding density of 150 × 10^3^ cells per well and transfected with the appropriate constructs (FHRE-Luc, E1Awt 12S) using Lipofectamine-2000 (Invitrogen) as recommended by the manufacturer. For stable plasmid integration, E1A + Ras cells were co-transfected with a vector coding the early region of Ad 5 (E1Aad5), and a selective pBABE-puro construct bearing the puromycin resistance gene. For the luciferase assay, cell extracts were prepared, and luciferase activity was determined according to the protocol supplied with the Luciferase Assay kit (Promega, Madison, WI, USA). Luciferase activity was determined using a TD-20/20 luminometer (Turner Designs, San José, CA, USA). Each experimental point was carried out in triplicate, and each experiment was performed no less than three times. Diagrams represent means ± SEM.

## 3. Results

### 3.1. NaBut-Dependent Modulation of FoxO1 Varies Depending on the Expression of E1A

**Figure 1:**
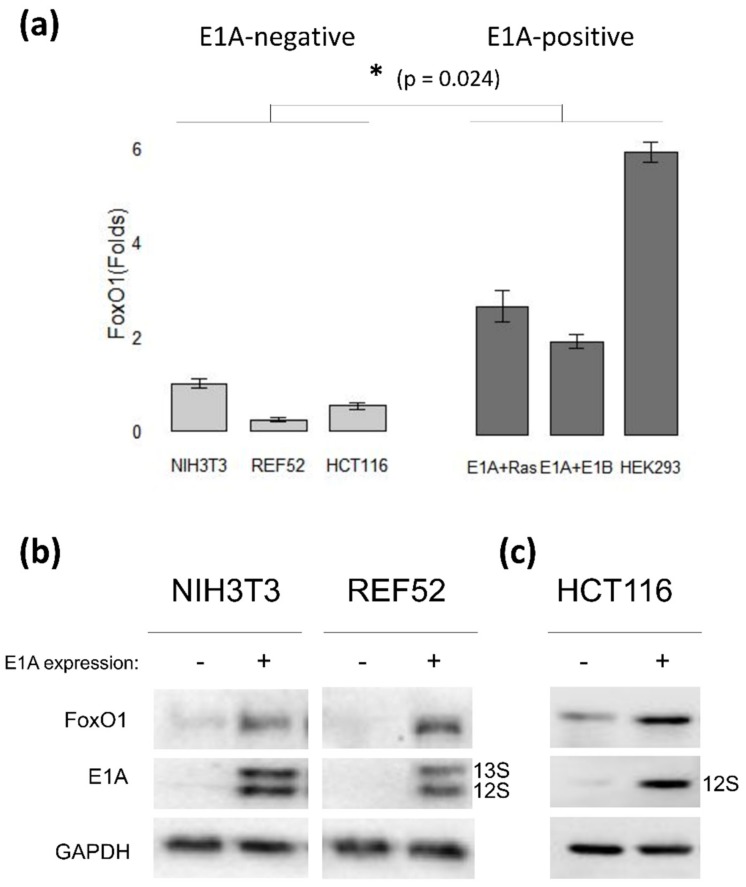
Early region 1A (E1A) overexpression causes the augmentation of the Forkhead box O (FoxO) protein level in rodent and human cells. (**a**) The plot represents the basal level of FoxO1 protein in E1A-negative (light gray) and E1A-expressing (dark gray) cell lines evaluated with immunoblotting, normalized against the basal FoxO1 level in NIH3T3 cells which was set at 1. Results are represented as a mean of 3–4 independent experiments; error bars indicate the standard error of the mean (SEM). The Mann–Whitney test was used to prove statistical significance of the difference between the basal FoxO1 level in E1A-negative and E1A-positive cells, marked with asterisk (*p* < 0.05). (**b**) NIH3T3 and REF52 cells were stably transfected with pE1A vector coding the early region of human adenovirus type 5 (E1Aad5). Cell extracts were analyzed by immunoblotting. (**c**) HCT116 cells were transfected with empty vector (−) or vector coding wild-type (wt) E1A 12S adenovirus protein (E1A). After 24 h, cells were lysed, and immunoblotting with antibodies to FoxO1 or E1A was performed. The plot represents FoxO1 expression levels, normalized against the basal FoxO1 level in non-transfected HCT116 cells.

Long-term HDACi treatment was shown to induce FoxO1 and FoxO3 degradation in E1A + Ras-transformed mouse cells [46]. In addition, we noticed that the level of E1A in these cells fell after 24 h of HDACi treatment. To test whether there is a link between these events, the influence of HDACi sodium butyrate (NaBut) on FoxO expression was analyzed in E1A-negative cells (NIH3T3 and REF52) and in cells expressing adenoviral E1A protein (E1A + Ras and E1A + E1B). The immunoblotting data demonstrate that the basal level of FoxO1 expression was higher in E1A-expressing cells compared to E1A-negative cells (Figure 1). To explore the effect of E1A on FoxO1 expression, we overexpressed E1A in E1A-negative rodent (NIH3T3, REF52) and human cancer cells (HCT116). Data in Figure 1b demonstrate the elevated basal levels of FoxO1 protein in mouse NIH3T3 and rat REF52 cells stably transfected with pE1A vector coding the early region of human adenovirus type 5 (E1Aad5). The same trend was observed in human tumor HCT116 cells, where the expression of E1A 12S induced an increase in FoxO1 expression (Figure 1c). Thus, we prove that the expression of E1A increases the stability of FoxO.

**Figure 2:**
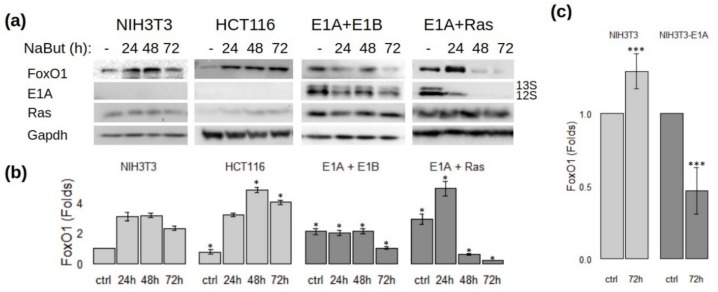
Sodium butyrate (NaBut) differently modulates FoxO1 expression in E1A-negative and E1A-positive cells. (**a**) NIH3T3, E1A + E1B, and E1A + Ras rodent cells and HCT116 human cells were treated with 4 mM NaBut for 24–72 h. Cell extracts were analyzed by immunoblotting. (**b**) The plots representing the load-control-weighted FoxO1 expression (fold change). Expression level was normalized against the expression level of the particular protein in the control sample of NIH3T3 cells which was set at 1. Single asterisk represents significant difference from the corresponding time point in NIH3T3 cells (*p* < 0.05). (**c**) The plots representing the FoxO1 expression in E1A-negative and E1A-expressing NIH3T3 cells (NIH3T3 stable transfected with pE1A vector coding the early region of Ad 5 (E1Aad5)) under prolonged NaBut treatment. Results are presented as fold changes in relation to the corresponding control sample. Triple asterisks represent significant difference between 72h points (*p* < 0.01).

Immunoblotting data reveal that NaBut induced FoxO1 accumulation in E1A-negative cells. On the contrary, in E1A-expressing cells, there was a tendency toward declining of the FoxO1 protein level after prolonged NaBut treatment (Figure 2a,b).

The expressions of E1A and FoxO1 correlated positively in E1A-expressing cells treated with sodium butyrate. NaBut-induced decrease of FoxO1 protein level was accompanied by a reduced expression of adenoviral protein E1A. The dynamic of FoxO1 level under NaBut treatment is shown more clearly on the plot (Figure 2b), representing the average band density, scaled to the control load and converted to the relative units—the ratio of weighted band density of a particular band to the control sample. Thus, we show that NaBut differently modulates FoxO1 expression in E1A-negative and E1A-positive rodent cells.

To test if the adenoviral E1A alters the effect of sodium butyrate on the expression of FoxO, we generated NIH3T3 cells transformed with plasmid pE1A containing the left end of the Ad5 genome (positions 1–1634) including the *E1A* gene and its endogenous promoter. The control NIH3T3 and E1A-expressing NIH3T3 cells were prolonged treated with NaBut (up to 72 h) and assayed for FoxO1 expression. Immunoblotting data demonstrate that prolonged action of sodium butyrate increased the initially low level of FoxO1 expression in E1A-negative cells NIH3T3. On the contrary, the initially elevated level of FoxO1 decreased in E1A-expressing cells under prolonged sodium butyrate treatment (Figure 2c).

### 3.2. The Adenovirus E1A Protein Expression Alters the Sodium Butyrate Influence on the Transactivation Function of FoxO

**Figure 3:**
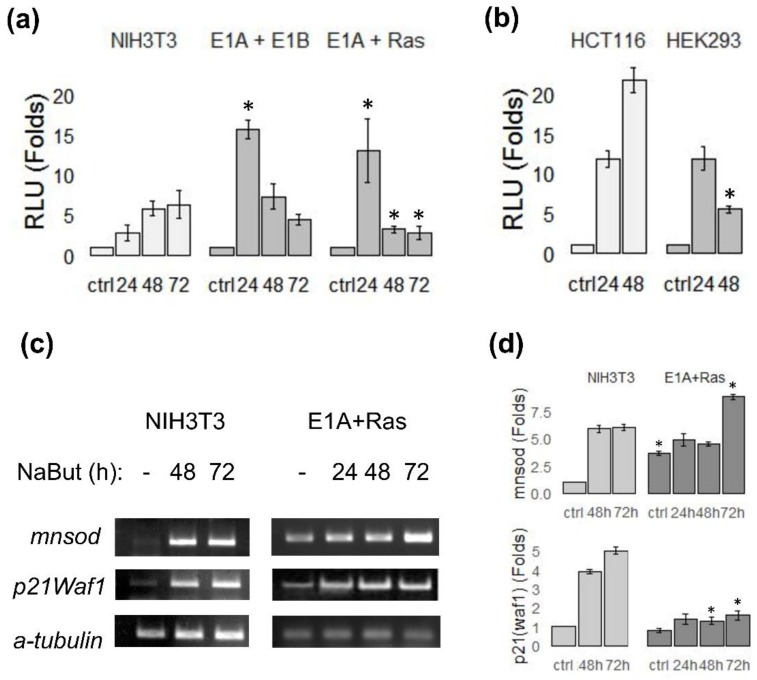
Sodium butyrate influence on FoxO activity in cells with different E1A status. The relative luciferase activity in rodent (**a**) and human (**b**) E1A-negative (NIH3T3, HCT116) and E1A-expressing (E1A + E1B, E1A + Ras or HEK293) cells transfected with FHRE-Luc reporter under FoxO-regulated promoter, untreated or treated with 4 mM NaBut for 24–72 h. Luciferase activity is represented in relation to corresponding control samples (untreated cells), which were set to 1. (**c**) Expression of FoxO target genes, evaluated using RT-PCR; the plots represent the dynamics of messenger RNA (mRNA) level in relation to corresponding control samples (set at 1). (**d**) The *alpha-tubulin*-weighted band density calculated based on RT-PCR results (**c**). Results are represented as a mean of 3–4 independent experiments; error bars indicate the standard error of the mean. The Mann–Whitney test was used to prove statistical significance of the difference in FoxO activity (**a**,**b**)/FoxO target gene expression (**d**) between corresponding time points in different cell lines in relation to E1A-negative cells (significant bars are marked with asterisks; *p* < 0.05).

To assess the influence of NaBut on the transactivation ability of FoxO in dependence of E1A expression, the ability of FoxO to activate the target gene transcription was analyzed in E1A-expressing and E1A-negative cells under prolonged NaBut treatment using RT-PCR and a luciferase assay. E1A-negative rodent NIH3T3 and E1A-expressing E1A + Ras and E1A + E1B cells were transfected with a vector, coding a luciferase under control of FoxO-responsive promoter (FHRE-Luc). Cells were left untreated or treated with NaBut for 24–72 h and assayed for luciferase activity. Luciferase assay data demonstrate that NaBut enhanced FoxO transactivity in both cell lines, but temporal dynamics in these cells were different (Figure 3a). NaBut steadily elevated the activity of luciferase in E1A-negative NIH3T3 cells over time. FoxO activity increased three-fold and six-fold after 24 and 72 h, respectively. By contrast, in E1A-transformants, the initial FoxO activation was followed by a significant activity decline under a longer NaBut administration (longer than 24 h). These data are consistent with the dynamics of FoxO1 protein, obtained with immunoblotting (Figure 2).

The same results were obtained in human cells. The luciferase activity in E1A-negative HCT116 cells kept increasing even after 48 h of NaBut treatment. FoxO activity increased 10-fold and 20-fold after 24 and 48 h, respectively. On the other hand, in E1A-expressing cells HEK293, FoxO-activity, after a temporary increase, decreased under a prolonged action of NaBut (Figure 3b).

We analyzed the expression of the FoxO target genes, *p21Waf1*, encoding the cyclin kinase inhibitor, and *MnSod*, the product of which is involved in the ROS inactivation, to validate the FoxO transactivation function changes under NaBut treatment. In general, the dynamics of FoxO target gene expression was consistent with the data obtained with a luciferase assay. RT-PCR analysis of the FoxO target gene expression revealed an increased basal level of mRNA in E1A-expressing cells (E1A + Ras), compared with E1A-negative cells (NIH3T3). Expression of the studied genes did not increase in E1A-expressing cells under prolonged exposure to sodium butyrate. On the other hand, in E1A-negative cells, an intense transcription increase was observed (Figure 3c).

Thus, the transactivating function of FoxO consistently increased with the time course of NaBut treatment in E1A-negative cells, whereas there was a tendency toward a decrease in FoxO activity under prolonged NaBut treatment in cells expressing the adenoviral E1A protein.

### 3.3. E1A Overexpression Stabilizes FoxO1 Protein and Further Enhances the FoxO Transactivation Ability under NaBut Treatment

**Figure 4:**
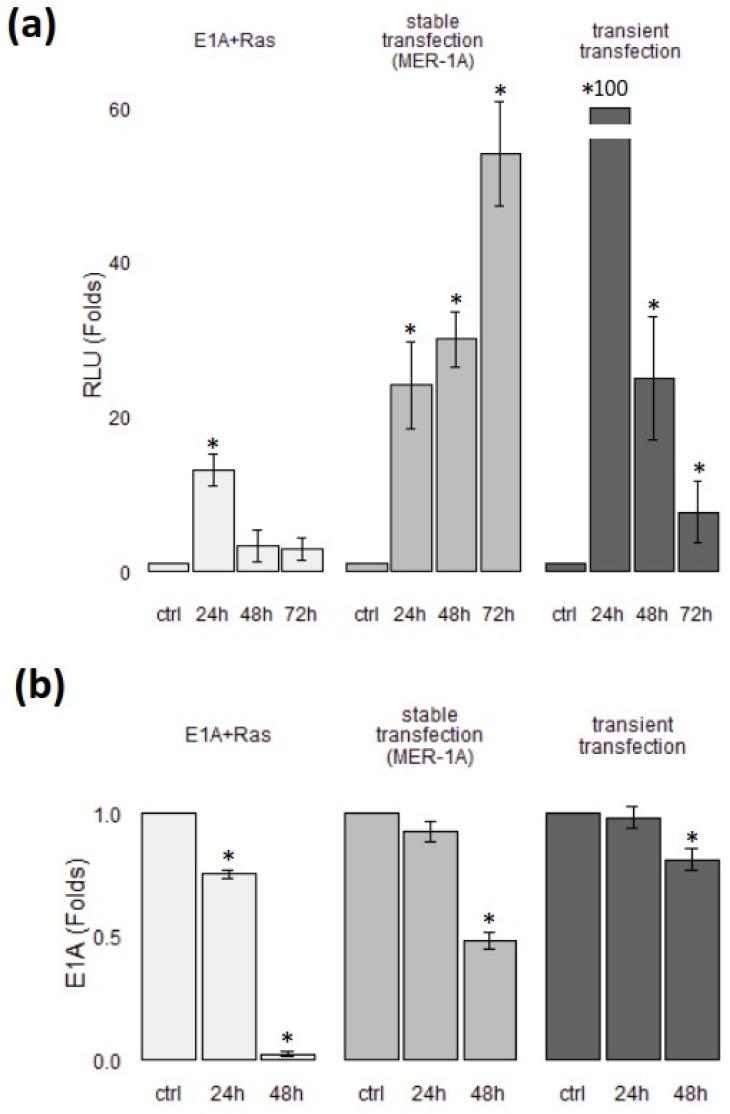
E1A overexpression rescues NaBut-dependent inhibition of FoxO activity in E1A + Ras cells. (**a**) The relative luciferase activity in E1A + Ras and MER-1A cells transfected with FHRE-Luc or in E1A + Ras cells co-transfected with FHRE-Luc and vector coding E1A 12S, untreated or treated with 4 mM NaBut for 24–72 h. (**b**) The plots representing the dynamics of E1A expression under NaBut treatment in cells described for previous experiments. Immunoblotting of cell extracts with antibodies against E1Aad5 in E1A + Ras, MER-1A, and E1A + Ras cells after additional E1A expression (stable and transient transfection), either untreated (ctrl) or treated with 4 mM NaBut for 24–48 h. E1A expression levels and FoxO activity are represented in relation to the control samples (untreated cells), which were set as 1. Single asterisk represents significant difference from baseline (*p* < 0.05).

We previously showed an HDACi-induced decrease in protein and mRNA level of E1A in HEK293 and E1A + Ras-transformed cells [14]. In E1A + Ras cells, the expression of the *E1A* gene is regulated by the native adenoviral promoter (adenovirus type 5) [47,48,49]. Thus, we evaluated whether the additional overexpression of E1A would further activate FoxO. In order to exclude the effect of NaBut on the *E1A* promoter, we performed a transient and stable transfection of E1A + Ras cells with the plasmid coding the 12S E1A under the control of a strong promoter of cytomegalovirus (CMV). The regulation and activity of CMV and Ad5 viral promoters differ significantly; thus, they can be used in target cells for different purposes. The high-activity CMV promoter is convenient for efficient transgene expression. Moreover, the CMV promoter was reported to be stimulated by HDACi [50]. Firstly, to verify the consequences of E1A overexpression on Foxo1 transactivation function, we obtained the stably transfected clones of E1A + Ras cells carrying an E1A under CMV promoter (MER-1A cell line). Then, MER-1A cells were transfected with a vector, coding luciferase under control of FoxO-responsive promoter (FHRE-Luc). Cells were left untreated or treated with NaBut for 24–72 h and assayed for luciferase activity. Figure 4a (middle bars) shows the averaged results of experiments obtained with the different clones of MER-1A. Luciferase assay data demonstrate that NaBut enhanced FoxO transactivation function over time in cells overexpressing E1A, implying that E1A overexpression abrogates NaBut-dependent inhibition of FoxO activity in E1A + Ras cells. Alternatively, E1A + Ras cells were co-transfected with the FHRE-Luc and CMV–E1A plasmid. The E1A overexpression under the strong CMV promoter multiplied both the basal FoxO activity (data not shown) and the NaBut-dependent increase of FoxO activity (Figure 4a). As shown in Figure 4a, NaBut increased the FoxO activity tenfold in E1A + Ras cells, while, in E1A + Ras cells with additional E1A overexpression, 50-fold or even 100-fold increases took place. However, FoxO activation after the transient E1A transfection (Figure 4a, dark-gray bars) was temporary, as it occurred in control cells (Figure 4a, light-gray bars), which may be possibly explained by lower vector stability in the transient assay. The immunoblotting of the transfected cell lysates was used to check the compatibility between FoxO activity and E1A protein level (Figure 4b).

Thus, our results suggest that, for overcoming NaBut-induced inhibition of the FoxO activity in E1A-expressing cells, increased expression of E1A independent of HDACi is required.

### 3.4. CBP/p300-Binding Domain of E1Aad5 Is Responsible for Stabilization of FoxO1 Protein

**Figure 5:**
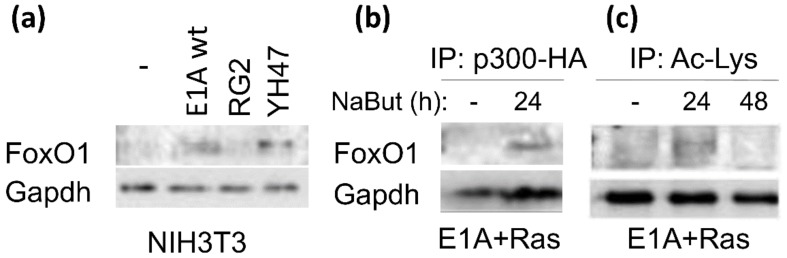
CBP/p300-binding domain of E1Aad5 is responsible for stabilization of FoxO1 protein. (**a**) Wild-type E1A 12S (wt) and Rb-binding mutant E1A 12S (YH47) induce FoxO1 protein accumulation in NIH3T3 cells. NIH3T3 cells were transfected with the adenovirus E1A constructs (E1A12S (wt), E1A12S (RG2), or E1A12S (YH47)). Cell extracts were analyzed by immunoblotting. (**b**) E1A + Ras cells were transfected with cytomegalovirus (CMV)-p300-hemagglutinin (HA) and treated with NaBut for 24 h. Cell extracts were immunoprecipitated with anti-HA antibody, followed by immunoblotting with FoxO1 antibody. (**c**) Cell extracts from E1A + Ras cells were immunoprecipitated with anti-acetylated lysine antibody and probed with anti-FoxO1 antibody.

To elucidate the mechanisms of E1A influence on the stability of FoxO, we tried to determine which regions of adenoviral E1A protein are important for the induction of FoxO activity. For this, NIH3T3 cells were transfected with different adenovirus E1A constructs. At 36 h after transfection, cells were lysed and assayed for FoxO1 expression. As shown in Figure 5a a plasmid expressing the wild-type (wt) E1A 12S induced FoxO1 protein accumulation in NIH3T3 cells. The E1A 12S (YH47) mutant encoding a protein that does not bind Rb and p130 stimulated FoxO1 expression with similar efficiency to wt E1A 12S (Figure 5a). The E1A 12S (RG2) mutant encoding a protein that does not bind p300 was unable to stimulate FoxO1 accumulation. These data indicate that the p300-binding region of the E1A protein plays the crucial role in the induction of FoxO.

Next, we investigated whether sodium butyrate modulates the interaction of p300 with FoxO in E1A + Ras cells. To assess the dynamic of FoxO/p300 interaction, E1A + Ras cells were transfected with a vector coding HA-tagged full-length p300 acetyltransferase, which was then treated with NaBut for 24 h. The coimmunoprecipitation assay followed with immunoblotting revealed that endogenous FoxO1 and p300 could associate in sodium butyrate-treated cells only (Figure 5b). In untreated control cells, the interaction of FoxO with p300 was prevented by competitive binding with the adenoviral protein E1A. This result is consistent with the data demonstrating the degradation of E1A protein under NaBut treatment (Figure 2a). To assess the effects of the NaBut-induced interaction of FoxO with p300, the level of FoxO1 acetylation was studied following NaBut treatment. Lysates of E1A + Ras cells were immunoprecipitated with anti-acetylated lysine antibody and probed with anti-FoxO1 antibody. As shown in Figure 5c, NaBut-induced augmentation of FoxO1 acetylation was followed by a significant acetylation decline under a longer NaBut administration. Prolonged inhibition of deacetylases by sodium butyrate in E1A + Ras cells did not further increase the amount of acetylated FoxO1 (Figure 5c). These data suggest that FoxO undergoes rapid degradation that follows protein acetylation, which is consistent with our data (Figure 2a).

## 4. Discussion

Our data show that the presence of adenoviral E1A significantly alters the cell response to NaBut treatment, specifically by influencing the dynamics of FoxO expression and activity. First of all, the basal level of FoxO1 expression in E1A-positive cells is generally higher. Moreover, we showed that both rodent (NIH3T3 and REF52) and human cancer cells (HCT116) transfected with a vector encoding the E1A are characterized by an increased basal level of FoxO1 protein. Secondly, our experimental data reveal that the transactivation function of FoxO is generally in line with the FoxO protein level. In E1A-negative cells, the low basal level of FoxO1 greatly increases under NaBut treatment, accompanied by enhanced FoxO transactivation function. As for E1A-expressing cells, there is a downward trend in FoxO1 expression and activity under prolonged NaBut treatment. Several mechanisms leading to FoxO stabilization by E1A protein are suggested. E1A can bind to the proteasomes and inactivate their functions [51,52], preventing the ubiquitin-dependent proteolysis of FoxO3a [23]. E1A also activates PP2A and MKP phosphatases [24,25], which abrogate the degradation of FoxO by reducing its phosphorylation [53,54]. Phosphorylation of FoxO1 by PKB/Akt kinase is an important regulator of FoxO1 activity and stability. Being phosphorylated with PKB/Akt, FoxO1 binds proteins 14-3-3, which leads to a decrease in its affinity to DNA, export from the cell nucleus, and retention in the cytoplasm. This implies that PKB/Akt is a negative regulator of FoxO1 activity [30,31]. E1A expression induces a decrease in both the basal and the induced status of PKB/Akt activity [29], which can also be one of the mechanisms of E1A-induced stabilization of FoxO1. This is consistent with our previous findings that HDACis mostly affect the FoxO1 on a protein level and do not lead to significant changes in FoxO1 transcription [46]. Thus, NaBut does not decline FoxO gene expression in E1A-positive cells, but rather facilitates degradation of the FoxO protein, drastically suppressing E1A expression. The direct binding of E1A to the acetyltransferases CBP (CREB binding protein) and p300 modulates CBP/p300 acetyltransferase activity [55]. At the same time, FoxOs transcription factors are the substrates for CBP/p300-mediated acetylation [56]. Thus, the competition between E1A protein and FoxO transcription factors for binding with CBP/p300 may act as another potential mechanism via which E1A can affect FoxO acetylation and stability. Acetylation of FoxO1 [56] and FoxO4 [57,58,59] attenuates their transcriptional activity. However, the mechanism underlying the negative effect of acetylation on FoxOs function remain to be elucidated. One explanation is that the positive charge of lysines in FoxOs contributes to their DNA-binding. Acetylation by CBP reduces FoxOs ability to bind DNA. Moreover, acetylation of FoxO1 increases its phosphorylation through the PI3K/PKB signaling pathways [60] and interaction with 14-3-3 proteins. Thus, competitive binding of the E1A protein to CBP/p300 acetyltransferases can prevent the interaction of CBP/p300 with FoxO, thereby preventing FoxO acetylation and destabilization.

We established the crucial role of the p300-binding region of the E1A protein in the induction of FoxO (Figure 5a). Comparison of the variants of mutant forms of E1A unable to bind pRb or p300 suggests that the p300-binding property is important for this stabilization. The p300 acetyltransferase, bound by E1A protein, is not able to interact with FoxO in control cells E1A + Ras (Figure 5b). HDACi-induced degradation of E1A leads to release of p300 and its binding to FoxO (Figure 5b). The subsequent p300-mediated acetylation of FoxO observed 24 h after NaBut treatment (Figure 5c) may act as a priming event for subsequent FoxO phosphorylation with activated PKB/Akt [60] and FoxO1 degradation. Therefore, we can suppose that the inhibition of the FoxO activity following NaBut treatment in E1A-expressing cells is due to the enhanced FoxO acetylation causing by the release of CBP/p300 from the complex with E1A.

In E1A-expressing cells, different dynamics of FoxO decrease are observed. In E1A + Ras cells, FoxO undergoes complete degradation, while the degradation of FoxO is less evident in E1A + E1B and HEK293 cells. This discrepancy may be attributed to the Ras oncoprotein. There are no Ras mutations in E1A + E1B and HEK293 cells [47], whereas E1A + Ras transformants contain activating mutations of the *cHa-ras* gene. Ras was shown to suppress FoxO1 expression, activating the Ras/PI3K/Akt pathway, leading to FoxO1 degradation [30,31]. As shown in Figure 1, the stable expression of E1A in the absence of Ras overexpression prevents FoxO1 from fast degradation in E1A + E1B and HEK293 cells. Our findings suggest that an exogenous E1A expression is insufficient for sustained FoxO activity independent of HDACi in E1A + Ras cells because HDACis induce degradation of E1A. However, the existence of E1A-independent mechanisms of FoxO suppression may not be rejected. We previously showed HDACi-induced extranuclear relocation of beta-catenin [61], which was proven to bind FoxO and increase its activity [62].

Our present study provides findings that HDACi-induced degradation of FoxO protein can be avoided, provided that the level of E1A protein does not decrease significantly following HDACi treatment. However, in cells expressing E1A under the adenoviral promoter, whose activity is inhibited by HDACi, the amount of E1A decreases following the addition of HDACi, which causes the FoxO expression and activity to decrease. In other words, expression of the early E1A region of adenovirus increases FoxO stability, but it makes the FoxO protein level more sensitive to HDACi treatment.

In summary, here, we present evidence showing that FoxO expression and stability under NaBut treatment is firmly associated with expression of E1A and Ras. Oncogenic Ras causes the degradation of FoxO through activation of the Ras/Akt signaling pathway, but expression of E1A in cells carrying activated Ras is able to overcome this inhibition and enhance FoxO expression.

The interpretation of these findings could be challenging since FoxOs are proven to cause both tumor suppression [44,63] and progression [41,43] depending on the context. Several lines of evidence suggest a critical role for FoxO members in the sensitivity of cancer cells to cytotoxic agents. Reduced expression of FoxO1 and FoxO3 is associated with resistance to conventional agents and with reduced efficacy of drug combinations in ovarian and breast cancer cells [64,65,66]. FoxOs play an important role in apoptosis by activating transcription of the ligand for the Fas-dependent cell death pathway *FasL*, and the proapoptotic Bcl-2 family member *Bim*. Alternatively, FoxO factors can promote cell-cycle arrest by activating the cell-cycle inhibitors *p21Waf1 or p27kip1* to induce G1 arrest, or *GADD45* to induce G2 arrest.

Our results suggest that, in E1A-expressing cells with a *Ras* gene mutation, long-term HDACi treatment inhibits FoxO expression and activity. We suggest that the E1A/HDACi co-treatment is ineffective in anticancer therapy of tumors with *Ras* mutations. On the other hand, there is a hypothesis that FoxO acts as a tumor suppressor in normal cells; however, within tumor cells, it can contribute to their survival and tumor growth [67,68]. If we presume this hypothesis to be correct, NaBut-induced FoxO degradation after prolonged treatment in E1A + Ras transformed cells seems to be expected outcome. However, we are yet to succeed in using HDACi to reduce FoxO level in human tumor cells, which can be a subject of further research.

## References

[1] Chow L.T., Broker T.R., Lewis J.B. (1979). Complex splicing patterns of RNAs from the early regions of adenovirus-2. J. Mol. Biol..

[2] Chinnadurai G. (2011). Opposing oncogenic activities of small DNA tumor virus transforming proteins. Trends Microbiol..

[3] Loewenstein P.M., Wu S.-Y., Chiang C.-M., Green M. (2012). The adenovirus E1A N-terminal repression domain represses transcription from a chromatin template in vitro. Virology.

[4] Pützer B.M., Stiewe T., Parssanedjad K., Rega S., Esche H. (2000). E1A is sufficient by itself to induce apoptosis independent of p53 and other adenoviral gene products. Cell Death Differ..

[5] Saito Y., Sunamura M., Motoi F., Abe H., Egawa S., Duda D.G., Hoshida T., Fukuyama S., Hamada H., Matsuno S. (2006). Oncolytic replication-competent adenovirus suppresses tumor angiogenesis through preserved E1A region. Cancer Gene Ther..

[6] Zhou Z., Zhou R., Guan H.J., Bucana C.D., Kleinerman E.S. (2003). E1A gene therapy inhibits angiogenesis in a Ewing’s sarcoma animal model. Mol. Cancer Ther..

[7] Liao Y., Yu D., Hung M.-C. (2007). Novel approaches for chemosensitization of breast cancer cells: The E1A story. Adv. Exp. Med. Biol..

[8] Chattopadhyay D., Ghosh M.K., Mal A., Harter M.L. (2001). Inactivation of p21 by E1A leads to the induction of apoptosis in DNA-damaged cells. J. Virol..

[9] Shao R., Lee D.-F., Wen Y., Ding Y., Xia W., Ping B., Yagita H., Spohn B., Hung M.-C. (2005). E1A sensitizes cancer cells to TRAIL-induced apoptosis through enhancement of caspase activation. Mol. Cancer Res..

[10] Sánchez-Prieto R., Quintanilla M., Cano A., Leonart M.L., Martin P., Anaya A., Ramón y Cajal S. (1996). Carcinoma cell lines become sensitive to DNA-damaging agents by the expression of the adenovirus E1A gene. Oncogene.

[11] Radke J.R., Siddiqui Z.K., Miura T.A., Routes J.M., Cook J.L. (2008). E1A oncogene enhancement of caspase-2-mediated mitochondrial injury sensitizes cells to macrophage nitric oxide-induced apoptosis. J. Immunol. Baltim..

[12] Yamaguchi H., Chen C.-T., Chou C.-K., Pal A., Bornmann W., Hortobagyi G.N., Hung M.-C. (2010). Adenovirus 5 E1A enhances histone deacetylase inhibitors-induced apoptosis through Egr-1-mediated Bim upregulation. Oncogene.

[13] Radke J.R., Siddiqui Z.K., Figueroa I., Cook J.L. (2016). E1A enhances cellular sensitivity to DNA-damage-induced apoptosis through PIDD-dependent caspase-2 activation. Cell Death Discov..

[14] Igotti M.V., Svetlikova S.B., Pospelov V.A. (2018). Overexpression of Adenoviral E1A Sensitizes E1A+Ras-Transformed Cells to the Action of Histone Deacetylase Inhibitors. Acta Nat..

[15] Burgess A., Ruefli A., Beamish H., Warrener R., Saunders N., Johnstone R., Gabrielli B. (2004). Histone deacetylase inhibitors specifically kill nonproliferating tumour cells. Oncogene.

[16] Minucci S., Pelicci P.G. (2006). Histone deacetylase inhibitors and the promise of epigenetic (and more) treatments for cancer. Nat. Rev. Cancer.

[17] Van Lint C., Emiliani S., Verdin E. (1996). The expression of a small fraction of cellular genes is changed in response to histone hyperacetylation. Gene Expr..

[18] Marks P.A., Richon V.M., Rifkind R.A. (2000). Histone Deacetylase Inhibitors: Inducers of Differentiation or Apoptosis of Transformed Cells. J. Natl. Cancer Inst..

[19] Karagiannis T.C., El-Osta A. (2006). Modulation of cellular radiation responses by histone deacetylase inhibitors. Oncogene.

[20] Lindemann R.K., Gabrielli B., Johnstone R.W. (2004). Histone-Deacetylase Inhibitors for the Treatment of Cancer. Cell Cycle.

[21] Marks P.A., Breslow R. (2007). Dimethyl sulfoxide to vorinostat: Development of this histone deacetylase inhibitor as an anticancer drug. Nat. Biotechnol..

[22] Kouzarides T. (1999). Histone acetylases and deacetylases in cell proliferation. Curr. Opin. Genet. Dev..

[23] Su J.-L., Cheng X., Yamaguchi H., Chang Y.-W., Hou C.-F., Lee D.-F., Ko H.-W., Hua K.-T., Wang Y.-N., Hsiao M. (2011). FOXO3a-Dependent Mechanism of E1A-Induced Chemosensitization. Cancer Res..

[24] Liao Y., Hung M.-C. (2004). A New Role of Protein Phosphatase 2A in Adenoviral E1A Protein-Mediated Sensitization to Anticancer Drug-Induced Apoptosis in Human Breast Cancer Cells. Cancer Res..

[25] Cimas F.J., Callejas-Valera J.L., Pascual-Serra R., García-Cano J., Garcia-Gil E., De la Cruz-Morcillo M.A., Ortega-Muelas M., Serrano-Oviedo L., Gutkind J.S., Sánchez-Prieto R. (2015). MKP1 mediates chemosensitizer effects of E1a in response to cisplatin in non-small cell lung carcinoma cells. Oncotarget.

[26] Singh A., Ye M., Bucur O., Zhu S., Tanya Santos M., Rabinovitz I., Wei W., Gao D., Hahn W.C., Khosravi-Far R. (2010). Protein Phosphatase 2A Reactivates FOXO3a through a Dynamic Interplay with 14-3-3 and AKT. Mol. Biol. Cell.

[27] Yan L., Lavin V.A., Moser L.R., Cui Q., Kanies C., Yang E. (2008). PP2A Regulates the Pro-apoptotic Activity of FOXO1. J. Biol. Chem..

[28] Wu Z., Jiao P., Huang X., Feng B., Feng Y., Yang S., Hwang P., Du J., Nie Y., Xiao G. (2010). MAPK phosphatase-3 promotes hepatic gluconeogenesis through dephosphorylation of forkhead box O1 in mice. J. Clin. Investig..

[29] Viniegra J.G., Losa J.H., Sánchez-Arévalo V.J., Cobo C.P., Soria V.M.F., Cajal S.R., Sánchez-Prieto R. (2002). Modulation of PI3K/Akt pathway by E1a mediates sensitivity to cisplatin. Oncogene.

[30] Brunet A., Bonni A., Zigmond M.J., Lin M.Z., Juo P., Hu L.S., Anderson M.J., Arden K.C., Blenis J., Greenberg M.E. (1999). Akt Promotes Cell Survival by Phosphorylating and Inhibiting a Forkhead Transcription Factor. Cell.

[31] Furukawa-Hibi Y., Kobayashi Y., Chen C., Motoyama N. (2005). FOXO transcription factors in cell-cycle regulation and the response to oxidative stress. Antioxid. Redox Signal..

[32] Chang Y.-W., Hung M.-C., Su J.-L. (2014). The anti-tumor activity of E1A and its implications in cancer therapy. Arch. Immunol. Ther. Exp. (Warsz.).

[33] Van der Heide L.P., Smidt M.P. (2005). Regulation of FoxO activity by CBP/p300-mediated acetylation. Trends Biochem. Sci..

[34] Chakravarti D., Ogryzko V., Kao H.Y., Nash A., Chen H., Nakatani Y., Evans R.M. (1999). A viral mechanism for inhibition of p300 and PCAF acetyltransferase activity. Cell.

[35] Carter M.E., Brunet A. (2007). FOXO transcription factors. Curr. Biol..

[36] Shankar S., Chen Q., Srivastava R.K. (2008). Inhibition of PI3K/AKT and MEK/ERK pathways act synergistically to enhance antiangiogenic effects of EGCG through activation of FOXO transcription factor. J. Mol. Signal..

[37] Klotz L.-O., Sánchez-Ramos C., Prieto-Arroyo I., Urbánek P., Steinbrenner H., Monsalve M. (2015). Redox regulation of FoxO transcription factors. Redox Biol..

[38] Burgering B.M.T., Kops G.J.P.L. (2002). Cell cycle and death control: Long live Forkheads. Trends Biochem. Sci..

[39] Accili D., Arden K.C. (2004). FoxOs at the crossroads of cellular metabolism, differentiation, and transformation. Cell.

[40] Hosaka T., Biggs W.H., Tieu D., Boyer A.D., Varki N.M., Cavenee W.K., Arden K.C. (2004). Disruption of forkhead transcription factor (FOXO) family members in mice reveals their functional diversification. Proc. Natl. Acad. Sci. USA.

[41] Gehling U.M., Ergün S. (2008). Mechanisms of tumour vascularisation. Memo Mag. Eur. Med. Oncol..

[42] Carmeliet P., Jain R.K. (2000). Angiogenesis in cancer and other diseases. Nature.

[43] Goto T., Takano M., Hirata J., Tsuda H. (2008). The involvement of FOXO1 in cytotoxic stress and drug-resistance induced by paclitaxel in ovarian cancers. Br. J. Cancer.

[44] Zhang H., Pan Y., Zheng L., Choe C., Lindgren B., Jensen E.D., Westendorf J.J., Cheng L., Huang H. (2011). FOXO1 inhibits Runx2 transcriptional activity and prostate cancer cell migration and invasion. Cancer Res..

[45] Sunters A., Fernández de Mattos S., Stahl M., Brosens J.J., Zoumpoulidou G., Saunders C.A., Coffer P.J., Medema R.H., Coombes R.C., Lam E.W.-F. (2003). FoxO3a transcriptional regulation of Bim controls apoptosis in paclitaxel-treated breast cancer cell lines. J. Biol. Chem..

[46] Morshneva A., Gnedina O., Svetlikova S., Pospelov V., Igotti M. (2018). Time-dependent modulation of FoxO activity by HDAC inhibitor in oncogene-transformed E1A+Ras cells. AIMS Genet..

[47] Graham F.L., Smiley J., Russell W.C., Nairn R. (1977). Characteristics of a Human Cell Line Transformed by DNA from Human Adenovirus Type 5. J. Gen. Virol..

[48] Pospelova T.V., Medvedev A.V., Kukushkin A.N., Svetlikova S.B., van der Eb A.J., Dorsman J.C., Pospelov V.A. (1999). E1A + cHa-ras transformed rat embryo fibroblast cells are characterized by high and constitutive DNA binding activities of AP-1 dimers with significantly altered composition. Gene Expr..

[49] Lowe S.W., Ruley H.E. (1993). Stabilization of the p53 tumor suppressor is induced by adenovirus 5 E1A and accompanies apoptosis. Genes Dev..

[50] Lai M.-D., Chen C.-S., Yang C.-R., Yuan S.-Y., Tsai J.-J., Tu C.-F., Wang C.-C., Yen M.-C., Lin C.-C. (2010). An HDAC inhibitor enhances the antitumor activity of a CMV promoter-driven DNA vaccine. Cancer Gene Ther..

[51] Turnell A.S., Grand R.J.A., Gorbea C., Zhang X., Wang W., Mymryk J.S., Gallimore P.H. (2000). Regulation of the 26S proteasome by adenovirus E1A. EMBO J..

[52] Zhang X., Turnell A.S., Gorbea C., Mymryk J.S., Gallimore P.H., Grand R.J.A. (2004). The Targeting of the Proteasomal Regulatory Subunit S2 by Adenovirus E1A Causes Inhibition of Proteasomal Activity and Increased p53 Expression. J. Biol. Chem..

[53] Kurimchak A., Graña X. (2014). PP2A: More than a reset switch to activate pRB proteins during the cell cycle and in response to signaling cues. Cell Cycle.

[54] Ablack J.N.G., Cohen M., Thillainadesan G., Fonseca G.J., Pelka P., Torchia J., Mymryk J.S. (2012). Cellular GCN5 Is a Novel Regulator of Human Adenovirus E1A-Conserved Region 3 Transactivation. J. Virol..

[55] Goodman R.H., Smolik S. (2000). CBP/p300 in cell growth, transformation, and development. Genes Dev..

[56] Daitoku H., Hatta M., Matsuzaki H., Aratani S., Ohshima T., Miyagishi M., Nakajima T., Fukamizu A. (2004). Silent information regulator 2 potentiates Foxo1-mediated transcription through its deacetylase activity. Proc. Natl. Acad. Sci. USA.

[57] Fukuoka M., Daitoku H., Hatta M., Matsuzaki H., Umemura S., Fukamizu A. (2003). Negative regulation of forkhead transcription factor AFX (Foxo4) by CBP-induced acetylation. Int. J. Mol. Med..

[58] Van der Horst A., Tertoolen L.G.J., de Vries-Smits L.M.M., Frye R.A., Medema R.H., Burgering B.M.T. (2004). FOXO4 is acetylated upon peroxide stress and deacetylated by the longevity protein hSir2(SIRT1). J. Biol. Chem..

[59] Dansen T.B., Smits L.M.M., van Triest M.H., de Keizer P.L.J., van Leenen D., Koerkamp M.G., Szypowska A., Meppelink A., Brenkman A.B., Yodoi J. (2009). Redox-sensitive cysteines bridge p300/CBP-mediated acetylation and FoxO4 activity. Nat. Chem. Biol..

[60] Matsuzaki H., Daitoku H., Hatta M., Aoyama H., Yoshimochi K., Fukamizu A. (2005). Acetylation of Foxo1 alters its DNA-binding ability and sensitivity to phosphorylation. Proc. Natl. Acad. Sci. USA.

[61] Abramova M.V., Pospelova T.V., Nikulenkov F.P., Hollander C.M., Fornace A.J., Pospelov V.A. (2006). G1/S arrest induced by histone deacetylase inhibitor sodium butyrate in E1A + Ras-transformed cells is mediated through down-regulation of E2F activity and stabilization of beta-catenin. J. Biol. Chem..

[62] Essers M.A.G., de Vries-Smits L.M.M., Barker N., Polderman P.E., Burgering B.M.T., Korswagen H.C. (2005). Functional Interaction Between ß-Catenin and FOXO in Oxidative Stress Signaling. Science.

[63] Greer E.L., Brunet A. (2005). FOXO transcription factors at the interface between longevity and tumor suppression. Oncogene.

[64] Lu H., Huang H. (2011). FOXO1: A potential target for human diseases. Curr. Drug Targets.

[65] Chakrabarty A., Bhola N.E., Sutton C., Ghosh R., Kuba M.G., Dave B., Chang J.C., Arteaga C.L. (2013). Trastuzumab-resistant cells rely on a HER2-PI3K-FoxO-survivin axis and are sensitive to PI3K inhibitors. Cancer Res..

[66] Beretta G.L., Corno C., Zaffaroni N., Perego P. (2019). Role of FoxO Proteins in Cellular Response to Antitumor Agents. Cancers.

[67] Charitou P., Rodriguez-Colman M., Gerrits J., van Triest M., Groot Koerkamp M., Hornsveld M., Holstege F., Verhoeven-Duif N.M., Burgering B.M. (2015). FOXOs support the metabolic requirements of normal and tumor cells by promoting IDH1 expression. EMBO Rep..

[68] Sykes S.M., Lane S.W., Bullinger L., Kalaitzidis D., Yusuf R., Saez B., Ferraro F., Mercier F., Singh H., Brumme K.M. (2011). AKT/FOXO signaling enforces reversible differentiation blockade in myeloid leukemias. Cell.

